# Strategic Insight into the Combination Therapies for Metastatic Colorectal Cancer

**DOI:** 10.3390/curroncol30070480

**Published:** 2023-07-07

**Authors:** Yoshihito Kano, Mitsukuni Suenaga, Hiroyuki Uetake

**Affiliations:** 1 Department of Clinical Oncology, Graduate School of Medical and Dental Sciences, Tokyo Medical and Dental University (TMDU), Tokyo 113-8510, Japan; kano.canc@tmd.ac.jp; 2 Department of Clinical Research, National Hospital Organization Disaster Medical Center, Tokyo 190-0014, Japan; 7110srg2@tmd.ac.jp

**Keywords:** combination therapy, metastatic colorectal cancer, molecular targeted therapy, conversion surgery, liquid biopsy, *RAS*, *KRAS^G12C^*

## Abstract

Colorectal cancer (CRC) is the second most common cause of cancer-related deaths worldwide. The 5-year survival rate after curative resection is almost 80%, however, it is still less than satisfactory for metastatic CRC (mCRC). The combination approach including surgery, chemotherapy, molecular targeted therapy, and immunotherapy is a promising strategy due to its synergistic anticancer effect. Moreover, circulating tumor DNA (ctDNA) analysis has been reported to stratify the post-operative risk of recurrence, thus providing clinically valuable information for deciding to conduct adjuvant chemotherapy. Furthermore, multiple new drugs that potentially target undruggable genes, including *KRAS,* have been developed. In this review, we discuss the current management of patients with mCRC and future perspectives in the light of a combination therapeutic strategy.

## 1. Introduction

Colorectal cancer (CRC) is the third most frequently occurring cancer and the second most common cause of cancer-related deaths worldwide [1]. Even though various therapeutic options have been developed so far, the prognosis of metastatic CRC (mCRC) still needs to be improved. Combination therapy including surgery, chemotherapy, molecular targeted therapy, and immunotherapy is a promising strategy due to its additive or synergistic anticancer effect [2]. Regarding chemotherapy, the backbone multidrug combination regimens, such as fluorouracil (5-FU), leucovorin (LV), plus oxaliplatin, FOLFOX regimen, and 5-FU/LV plus irinotecan, FOLFIRI regimen, have been established in mCRC. Moreover, molecularly targeted drugs, such as an anti-vascular epidermal growth factor (VEGF) antibody or anti-epidermal growth factor receptor (EGFR) antibody plus backbone regimen, have been proven to improve clinical efficacy [3]. Furthermore, the combination of therapy with surgery and chemotherapy has also drawn attention. For example, while postoperative adjuvant chemotherapy after hepatectomy is still controversial, perioperative chemotherapy plus targeted therapy demonstrated its efficacy for patients with liver metastases [4,5]. Intriguingly, very recently, it has been reported that circulating tumor DNA (ctDNA) analysis could stratify patients at postsurgical risk of recurrence and this could be useful information for deciding to conduct adjuvant chemotherapy [6].

New targeted therapies have improved the outcomes of molecularly selected mCRC patients with a human epidermal growth factor receptor 2 (*HER2*) gene amplification, *BRAF^V600E^* mutation, and deficient mismatch repair (dMMR)/microsatellite instability(MSI)-high [7]. Of note, *RAS* had been an undruggable target for a while, however, a compound that targets a specific activating mutation, *KRAS^G12C^*, has been reported [8]. The compound exploits the reactive cysteine, 12C, and binds to it irreversibly, while not recognizing or inhibiting the wild-type *KRAS*, and therefore thought to act specifically on cancer cells harboring *KRAS^G12C^*. The inhibitors are currently approved for lung cancer patients and are under clinical trials in mCRC patients. Importantly, these agents are less than satisfactory when used in monotherapy, especially for patients with mCRC, therefore, the combination strategy is crucial for better response. 

Thus, systemic chemotherapy for mCRC has been greatly improved, however, the prognosis of mCRC still has room for improvement in combination with chemotherapy and/or molecular-targeted drugs, and immunotherapy with a biomarker-based approach. This review aims to summarize the systemic therapy for mCRC focusing on the combination treatment. 

## 2. Current Combination Therapies

### 2.1. Multidrug Combination Treatment

Due to the development of novel chemotherapeutic agents, including molecular targets and multidrug combination therapies, colorectal cancer (CRC) treatment has been greatly advanced, resulting in better response rates and longer survival time. The backbone multidrug combination regimens, such as fluorouracil (5-FU), leucovorin (LV), plus oxaliplatin, FOLFOX, and 5-FU/LV plus irinotecan, and FOLFIRI have been established in CRC. 5-FU/LV can be replaced by oral drugs, S-1 or capecitabine and S-1 plus oxaliplatin (SOX), capecitabine plus oxaliplatin (CapeOX), S-1 plus irinotecan (IRIS) and capecitabine plus irinotecan (CapeIRI) have been widely recognized. Moreover, the FOLFOXIRI regimen showed a superior response rate (34% versus 60%, *p* < 0.0001), progression-free survival, and overall survival rate (median PFS, 6.9 vs. 9.8 months; hazard ratio [HR], 0.63; *p* = 0.0006; and median OS, 16.7 vs. 22.6 months; HR, 0.70; *p* = 0.032) compared to the FOLFIRI regimen [9]. These combination therapies have clinical efficacy, however, can also cause adverse events, such as neurotoxicity or diarrhea. In particular, the FOLFOXIRI regimen increased grade 3 to 4 neutropenia. Therefore, selecting the regimen needs to be considered whether each case can be fit or vulnerable to the treatment options.

Bevacizumab is a humanized recombinant monoclonal antibody that blocks all isoforms of VEGF-A. Bevacizumab showed efficacy when added to first-line oxaliplatin-based chemotherapy in patients with metastatic CRC. Improved PFS was observed (9.4 vs. 8.0 months; and HR, 0.83; *p* = 0.0023) in comparison to the placebo group, although OS and RR were not significantly improved by the additional combination with bevacizumab [10]. Cetuximab and panitumumab are monoclonal antibodies that specifically target EGFRs and inhibit the activity of downstream signaling. The additional efficacy of cetuximab was observed with significantly improved RR (61% vs. 37%; *p* = 0.011) in the patients with *KRAS* wild-type (WT) at the first-line setting [11]. Furthermore, the PRIME study demonstrated that the addition of panitumumab to the FOLFOX regimen significantly improved PFS compared with FOLFOX alone (median PFS, 9.6 vs. 8.0 months; and HR, 0.80; *p* = 0.02) in *KRAS^WT^* patients [12]. Similarly, the phase III CRYSTAL study showed that the addition of cetuximab to FOLFIRI as first-line treatment significantly improves the PFS in patients with *KRAS^WT^* [13].

### 2.2. Which Is Better for the First Line, Cetuximab/Panitumumab or Bevacizumab in RAS^WT^ Tumors?

As described above, anti-EGFR antibodies (cetuximab or panitumumab) and an anti-VEGF antibody (bevacizumab) have both been shown to provide additional clinical outcomes in mCRC patients in combination with chemotherapy. However, their comparative treatment efficacy as the first-line setting has been controversial. In a randomized phase III FIRE-3 study, FOLFIRI plus cetuximab or FOLFIRI plus bevacizumab were compared for first-line treatment, resulting in better median OS in the cetuximab group (OS, 28.7 months vs. 25.0 months; and HR, 0.77; *p* = 0.017) in patients with *KRAS* codon12/13 WT mCRC. Importantly, the retrospective pooled analysis, including six randomized trials (CRYSTAL, FIRE-3, CALGB 80405, PRIME, PEAK, and 20050181), demonstrated that a significant clinical benefit of chemotherapy plus EGFR antibody over chemotherapy alone or chemotherapy plus bevacizumab was observed in patients with *RAS^WT^* (*KRAS* exon 2–4 WT; *NRAS* exon 2–4 WT) and left-sided tumors [14]. Very recently, in a phase III PARADIGM study, first-line chemotherapy in combination with panitumumab significantly improved overall survival among patients with left-sided *RAS^WT^* mCRC compared to the bevacizumab group (median OS, 37.9 months vs. 34.3 months; and hazard ratio for death, 0.82) [15]. Notably, in the subgroup analysis, the significant difference in OS between the panitumumab and bevacizumab groups was not observed in right-sided tumors. Because primary tumors arising from the left and right sides of the colon have distinct molecular characteristics, including *BRAF*, *HER2*, or microsatellite instability (MSI) and there is no information regarding molecular status in this study, further investigation will be necessary to understand the different sensitivity to EGFR and VEGF antibodies. Also recently, in an open-label, multicenter, randomized, phase III CAIRO5 study, FOLFOXIRI plus bevacizumab regimen demonstrated superior clinical benefit over FOLFOX/FOLFIRI plus bevacizumab in patients with initially unresectable liver metastasis and a right-sided *RAS^Mut^* or *BRAF^V600E^* mCRC [16]. 

### 2.3. Novel Targeted Therapies

#### 2.3.1. Targeting ERBB2 with Multiple Antibodies

**Figure 1:**
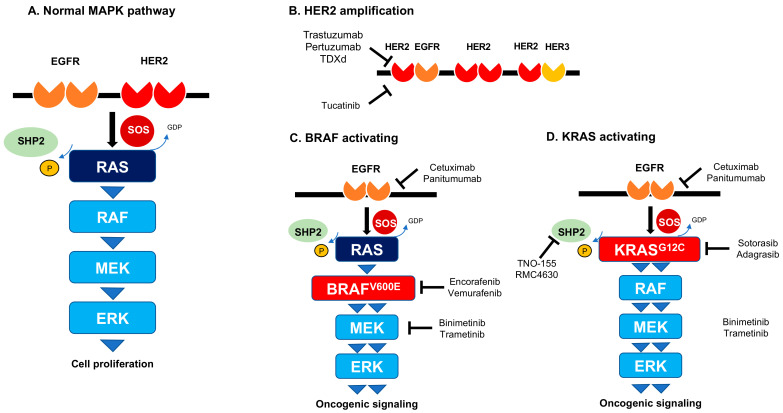
EGFR-MAPK signaling pathway and the targeted point by specific inhibitors. (**A**) Normal MAPK pathway. (**B**) HER2 amplification. HER2 forms homodimer or couples with heterodimeric partners, EGFR or HER3. (**C**) BRAF activating signaling. A triple combination with BRAF, MEK, and EGFR inhibition demonstrated significant clinical benefit. (**D**) KRAS activating signaling be specific mutation at G12C. The dual inhibition of *KRAS^G12C^* and EGFR demonstrated a better response. The addition of SHP2 inhibition might overcome the resistance of the *KRAS^G12C^* inhibitor.

The *HER2* (human epidermal growth factor receptor 2) or *ERBB2* is a proto-oncogene that encodes for a transmembrane glycoprotein receptor with tyrosine kinase activity. *HER2* lacks ligand-binding activity and its signaling function is engaged by its ligand-bound heterodimeric partner *HER3*, encoded by *ERBB3* [17]. In CRC, *HER2*-activating mutation showed resistance to cetuximab and panitumumab by sustaining MAPK signaling (Figure 1A) and was thus deemed as a negative predictive biomarker [18]. Similarly, *HER2* amplification has been reported to be a negative biomarker for the response to anti-EGFR antibody therapy and screening test for *HER2* should be considered before the treatment in mCRC patients [19,20]. A recent report showed that approximately 4–5% of CRCs are positive for *HER2* amplification or short variant mutation [19]. Targeting *HER2* in breast and gastric cancer has been successfully developed and antibodies, such as trastuzumab, pertuzumab, ado-trastuzumab emtansine, and trastuzumab deruxtecan (T-DXd) as well as pan-ERBB small molecule inhibitors, such as lapatinib or afatinib, exhibit clinical efficacy against these types of cancers [19]. Therefore, *HER2* has been an emerging target for *HER2*-positive CRC. Phase II trials, HERACLES and MyPathway studies demonstrate the clinical benefit with objective response rates (ORR) of 30% to 38% for patients with *HER2*-overexpressing CRC who are treated with a combination of trastuzumab plus lapatinib or trastuzumab plus pertuzumab, respectively [21,22]. It is noted that *KRAS* mutation showed lower ORR, shorter median PFS, and OS compared to *KRAS^WT^* in the subgroup analysis of the MyPathway study. Notably, a phase II trial, TRIUMPH study showed the promising efficacy of combination therapy with trastuzumab and pertuzumab for *RAS^WT^* CRC harboring *HER2* amplification confirmed by tissue and/or ctDNA [23]. The study demonstrated that ORR was 30% (8/27) in tissue-positive patients and 27% (7/25) in ctDNA-positive patients, which led to the first approval for trastuzumab plus pertuzumab in *HER2*-positive CRC in Japan in 2022. Furthermore, a global phase II trial, MOUNTAINEER study has reported that confirmed ORR was 38.1% including 3 patients of CR and 29 patients of PR in a combination with trastuzumab and tucatinib, a highly selective and oral tyrosine kinase inhibitor for *HER2* [24,25]. This result led to the accelerated FDA approval for *HER2*-positive mCRC in 2023. Furthermore, T-DXd has recently shown solid results with 45.3% of ORR, 6.9 months of PFS, and 15.5 months of OS in the patients with *HER2*-positive (immunohistochemistry, IHC3+ or IHC2+/in situ hybridization, ISH+) CRC in DESTINY-CRC01 study. Of note, no clinical response was observed in *HER2*-negative (IHC2+/ISH− and IHC1+) patients [26]. Thus, combination therapies, including a newly developed HER2 inhibitor deliver clinical benefits to mCRC patients harboring *HER2* amplification and further multiple clinical trials are ongoing and show promising data (Figure 1B).

#### 2.3.2. Targeting BRAF in Combination with Upstream Blocking

*BRAF* gene encodes a cytoplasmic serine/threonine-protein kinase that plays a crucial role in regulating the mitogen-activated protein kinase (MAPK) pathway. *BRAF* somatic missense mutations were observed in over 60% of malignant melanomas and at lower frequency in a wide range of human cancers [27]. *BRAF ^V600^* mutation occurs in approximately 10% of mCRC with a particularly poor prognosis [28]. A highly selective oral inhibitor of the BRAF V600 kinase, vemurafenib is associated with a high response rate of approximately 50% and improved survival in patients with malignant melanoma harboring *BRAF^V600E^* [29]. However, a histology-independent phase II basket study demonstrated that no responses were observed in the cohort of patients with mCRC who received vemurafenib monotherapy [30]. Preclinical studies suggest that the lack of efficacy of BRAF inhibitor in *BRAF^V600E^* mCRC is due to adaptive feedback reactivation of EGFR-mediated MAPK signaling. The clinical trial demonstrated that triple inhibition with BRAF (dabrafenib), EGFR (panitumumab), and MEK (trametinib) improved the clinical efficacy with 21% of ORR while double inhibition with BRAF and EGFR or EGFR and MEK showed an ORR of 10% and 0%, respectively [31]. Finally, a phase III trial, BEACON study has provided a shred of strong evidence that a triple combination with encorafenib, binimetinib, and cetuximab resulted in significantly longer overall survival (9.0 months) and a higher response rate (26%) than cetuximab plus chemotherapy-based standard therapy [32], leading to its U.S. FDA breakthrough treatment designation in April 2018. Notably, the updated data have shown that the median OS was 9.3 months for triplet, 9.3 months for doublet (cetuximab plus encorafenib), and 5.9 months for standard therapy; confirmed ORR was 26.8% for triplet, 19.5% for doublet and 1.8% for standard therapy [33], which led the U.S. FDA and European Medicines Agency (EMA) to approve the doublet combination with encorafenib and cetuximab for previously treated patients with mCRC harboring *BRAF^V600E^* in April 2020. Subgroup analyses suggested that patients with high baseline levels of CRP, ECOG performance status of 1, incompletely resected primary tumor, and more than two organs involvements appeared to favor triplet therapy relative to doublet therapy [33]. Of note, the phase II ANCHOR CRC study has been reported very recently and this trial showed a clinical benefit with 47.4% of ORR, 5.8 months of median PFS, and 18.3 months of median OS for triple combination (encorafenib, binimetinib, and cetuximab) in previously untreated *BRAF^V600E^*-positive CRC patients [34]. Also, in the first-line setting for *BRAF^V600E^* mCRC, a randomized, open-label phase II FIRE-4.5 trial investigated the efficacy of FOLFOXIRI in combination with cetuximab or bevacizumab. FOLFOLFOXIRI plus cetuximab did not demonstrate better outcome compared to FOLFOXIRI plus bevacizumab and thus bevacizumab-based triplet chemotherapy is still considered to be a recommended regimen for previously untreated *BRAF^V600E^* mutant mCRC patients. Further investigation of the first-line treatment among a triple combination (encorafenib, binimetinib, and cetuximab) or bevacizumab-based triplet chemotherapy will be needed. Further combination of other targeted drugs or chemotherapy with the doublet regimen as a backbone will be promising for additional efficacy (Figure 1C).

#### 2.3.3. Combination Immunotherapy for Patients with DNA Mismatch Repair-Deficient (dMMR)/Microsatellite Instability-High (MSI-H)

mCRC patients exhibit approximately 5% of dMMR/MSI-H which is a predictive biomarker for response to immunotherapies, such as anti-programmed death-1 (PD-1) checkpoint inhibitor or cytotoxic T-cell lymphocyte antigen-4 (CTLA-4) checkpoint inhibitor [35]. A fully human monoclonal antibody that inhibits PD-1, nivolumab monotherapy, showed a good clinical response with 31% of ORR, 50% of 1-year PFS, and 73% of 1-year OS in previously treated patients with dMMR/MSI-H [36]. Similarly, in KEYNOTE-164, an anti-PD1 humanized monoclonal antibody, pembrolizumab monotherapy, provided 33% of ORR in pretreated patients with MSI-H mCRC [37]. Nivolumab and ipilimumab (fully human monoclonal antibody inhibitor of CTLA-4) synergistically promote T-cell antitumor activity and thus a combination of nivolumab and ipilimumab in previously treated mCRC patients with dMMR/MSI-H demonstrated a better response with 55% of ORR, 71% of 1 year-PFS and 85% of 1 year-OS with manageable treatment-related adverse events (AEs) compared to nivolumab monotherapy [38]. Furthermore, a combination of nivolumab plus ipilimumab as a first-line setting in dMMR/MSI-H mCRC patients demonstrated promising clinical outcomes with 84% of disease control rate (DCR) [39]. The results of clinical trials for targeted therapies and immunotherapies are summarized in Table 1. 

**Table 1:** Clinical trials with targeted therapies and immunotherapies in metastatic colorectal cancer.

Target	Trial	Phase	No. of Patients	Regimen	Line of Treatment	ORR (%)	mPFS	mOS
HER2	HERACLES-A [21]	II	27	Trastuzumab + lapatinib	Late Lines	30	5.3 Mo	11.5 Mo
	MyPathway [22]	II	37	Trastuzumab + pertuzumab	Late Lines	38	4.6 Mo	10.3 Mo
	TRIUMPH [23]	II	19	Trastuzumab + pertuzumab	Late Lines	35	4 Mo	-
	MOUNTAINEER [24,25]	II	84	Trastuzumab + tucatinib	Late Lines	38.1	8.1 Mo	18.7 Mo
	DESTINY-CRC01 [26]	II	53	Trastuzumab deruxtecan	Late Lines	45.3	6.9 Mo	15.5 Mo
BRAF V600E	NCT00405587 [40]	II	21	Vemurafenib	2nd/late lines	5	2.1 Mo	7.7 Mo
	NCT01750918 [31]	I/II	20	Dabrafenib + panitumumab	1st/2nd	10	3.5 Mo	13.2 Mo
			91	Dabrafenib + trametinib + panitumumab	1st/2nd	21	4.2 Mo	9.1 Mo
	BEACON [32,33]	III	220	Encorafenib + cetuximab	2nd/late lines	19.5	4.3 Mo	9.3 Mo
			224	Encorafenib + binimetinib + cetuximab	2nd/late lines	26.8	4.5 Mo	9.3 Mo
MSI/dMMR	KEYNOTE-164 [37]	II	61	Pembrolizumab	Late Lines	33	2.3 Mo	31.4 Mo
	KEYNOTE-177 [41]	III	153	Pembrolizumab	1st	43.8	16.5 Mo	-
	CheckMate-142 [36,39]	II	74	Nivolumab	Late Lines	31	50% (1-year)	73% (1-year)
			119	Nivolumab + ipilimumab	Late Lines	55	71% (1-year)	85% (1-year)
			45	Nivolumab + ipilimumab	1st	69	76% (1-year)	84% (1-year)

## 3. Multidisciplinary Treatment for Metastatic Lesions

### 3.1. Combination Therapy of Surgical Resection and Chemotherapy for Liver Metastases

The 5-year survival rate after stage I–III CRC curative resection is almost 80%. However, it is only 13% for stage IV CRC, which accounts for 15–18% of all CRC. Moreover, almost 60% of patients with stage IV CRC develop liver metastases and hepatic recurrence occurs in 9–13% of all CRC after curative resection [4]. Thus, the strategy for hepatic metastases of CRC needs to be reconstructed and some important clinical studies have been conducted to evaluate the liver resection rate using multidisciplinary therapy combined with surgical resection and chemotherapy including molecular targeted drugs. It has been unclear whether adjuvant chemotherapy after hepatectomy could be clinically beneficial in liver-only metastatic colorectal cancer (CRC). In a phase II/III trial (JCOG0603) conducted in Japan, patients with an unlimited number of liver metastases were randomly assigned to hepatectomy alone or combination therapy with post-chemotherapy (12 courses of adjuvant mFOLFOX6) and hepatectomy. The results showed that the addition of adjuvant chemotherapy improved the 5-year DFS (49.8% vs. 38.7%; and HR, 0.67; *p* = 0.006) while improvement in the 5-year OS was not observed (71.2% vs. 83.1%) [42]. Thus, postoperative adjuvant chemotherapy after hepatectomy is still controversial; therefore, perioperative chemotherapy to control microscopic metastases is crucial. The combination of perioperative chemotherapy and surgery was compared to surgery alone for patients with initially resectable liver metastases. The study reported that a total of 12 cycles of preoperative and postoperative FOLFOX4 therapy improved the rate of PFS at 3 years [43]. Regarding conversion therapy with the intent of down-sizing the tumor burden and permitting surgical resection [44], Adam et al. demonstrated that the patients with unresected colorectal liver metastases who responded to chemotherapy underwent liver surgery and a 5-year survival of 33% was achieved [45]. Subset analysis from CALGB/SWOG 80405 trial showed that the patients who reached no evidence of disease (NED) after chemotherapy and surgery had long-term survival (>5 years) [46]. The PRIME study showed that the complete resection rate after the treatment of panitumumab-FOLFOX4 or FOLFOX4 alone was 8.3% and 7.0%, respectively [12]. A randomized phase II trial CELIM study that was conducted to evaluate the response to the treatment of cetuximab plus FOLFOX or FOLFIRI in CRC patients with unresectable liver metastases demonstrated good conversion rates with R0 resection of 38% and 30%, respectively [47]. Thus, these studies indicate that conversion therapy offers a clinical benefit. However, the criteria for resection of liver metastases differ between surgeons and the postoperative recurrence rate is still high [48]. Moreover, hepatic metastasis of degree H3 stipulated by the Japanese classification of colorectal carcinoma [49] is one of the poor prognosis factors [50]. Therefore, a multicenter joint phase II TRICC-0808 and ATOM trials were conducted to assess a preoperative therapy for CRC patients with H2/H3 liver metastases in Japan. The TRICC-0808 study demonstrated that RR was 46.2% and DCR was 92.4%. Moreover, conversion surgery after bevacizumab plus chemotherapy was achieved for 23.1% of patients during the protocol treatment [4]. The final analysis reported that the 3-year OS rate in patients with hepatectomy including resection after additional chemotherapy significantly improved the median survival rate (MST) compared to patients without hepatectomy (OS rate, 61.3% vs. 0%; and MST, 43.1 months vs. 21.0 months; *p* < 0.0001) [5]. Furthermore, a phase II ATOM study has compared the outcomes of chemotherapy plus bevacizumab versus cetuximab in CRC patients with initially unresected liver metastases. While cetuximab achieved a better response rate (84.7% vs. 68.4%) compared to the bevacizumab group, especially for patients with fewer but larger liver metastases, median PFS was not significantly improved (14.8 months vs. 11.5 months; and HR, 0.803; *p* = 0.33) and overall resection rate was similar (56.1% vs. 49.2%) [51]. Therefore, both combination therapies are still viable treatment options for liver metastases (Table 2). 

**Table 2:** Clinical trials with the response to chemotherapies for unresectable colorectal liver metastases.

Reference	Regimen	Phase	RR	Resection Rate	PFS	OS	No. of Patients	Liver Metastases
Adam et al. [43]	FOLFOX/FOLFIRI	-	-	12.5	-	33% (5-year)	1104	75%
CALGB/SWOG 80405 [44]	Chemo + BV	III	57	67	-	67.4	75	53.30%
	Chemo + Cet		66	78	-	64.1	105	50%
CELIM [45]	FOLFOX + Cet	II	68	38	10.8	53.9 * vs. 21.9 (*p* < 0.001)	56	100%
	FOLFIRI + Cet		57	30	10.5		55	
TRICC-0808 [4,5]	mFOLFOX6 + BV	II	46.2	44.4	-	43.1 * vs. 21.0 (*p* < 0.0001)	46	100%
ATOM [49]	mFOLFOX6 + BV	II	68.4	56.1	11.5 Mo	-	61	100%
	mFOLFOX6 + Cet		84.7	49.2	14.8 Mo	-	61	
Tomasello et al. [52]	FOLFOXIRI + BV	Meta- analysis	69	39.1	12.4	30.2	889	18–100%

* Comparison with resection vs. without resection.

### 3.2. Management of Peritoneal Metastases and Pulmonary Metastases

In general, peritoneal metastatic CRC is associated with a poor prognosis. Analysis and Research in Cancers of the Digestive System (ARCAD) database revealed that a median OS was shorter in patients with peritoneal metastasis compared to liver and lung metastasis (16.3 vs. 19.1 vs. 24.6 months, and HR, 0.79 and 0.61) [53]. Uni- and multivariate analyses of the retrospective cohort study demonstrated that postoperative chemotherapy and the regimen of chemotherapy were not included in factors affecting prognosis after R0 resection in patients with synchronous peritoneal metastatic CRC [54]. From the analysis of a prospectively expanded single-institutional database with over 2400 CRC cases, systemic chemotherapy tended to improve the median survival (17.9 vs. 7.03 months, *p* = 0.054) compared to the era of treatment without chemotherapy or only 5-Fluorouracil [55]. Furthermore, the addition of bevacizumab into first-line chemotherapy improved overall median survival (11 vs. 7.5 months, and HR, 0.7) in patients with peritoneal metastasis only and extraperitoneal metastases including liver or lung [56]. On the other hand, the subanalysis of two randomized controlled trials, including CAIRO and CAIRO2, suggested a poor prognosis for the patients with peritoneal metastatic CRC compared to those without peritoneal metastases in treatment with systemic chemotherapy with or without targeted therapy [57]. Thus, the same strategy as advanced CRC without peritoneal metastases should be considered for patients with peritoneal metastatic CRC, although the efficacy remains unsatisfactory. Novel combination strategies with immune checkpoint blockade, such as regorafenib plus ipilimumab and nivolumab [58] or botensilimab plus balstilimab [59] are being developed in phase I trial for microsatellite stable mCRC, including peritoneal metastases. 

As for lung metastases, surgical resection has been considered to be a standard treatment option for CRC patients. However, the efficacy of peri-operative chemotherapy after lung metastasectomy remains controversial. The meta-analysis including eight studies demonstrated that peri-operative chemotherapy, such as oxaliplatin- or irinotecan-containing regimens had a clinical benefit with better OS (HR, 0.83; *p* < 0.05) and PFS/RFS/DFS (HR, 0.67; *p* < 0.05) in patients after surgical resection [60]. In the future, large and prospective studies are needed to validate the role of adjuvant chemotherapy after lung metastasectomy in mCRC patients with lung metastases.

### 3.3. Combination with Liquid Biopsy to Improve the Efficacy of Adjuvant Chemotherapy

Recently, comprehensive genomic profiling (CGP) using next-generation sequencing (NGS) has been utilized worldwide in clinical settings to pursue precision medicine for advanced cancers. In general, CGP is performed using tissue specimens, however, liquid biopsy using circulating tumor DNA (ctDNA) from plasma has become clinically available. Liquid biopsy is a versatile and noninvasive tool to characterize genomic alterations without requiring invasive tissue biopsy [61]. Increasing numbers of studies indicate that liquid biopsy can identify targetable mutations, which is associated with better clinical outcomes in solid cancers, including CRC [62,63]. A large prospective GALAXY study has recently been conducted that monitors ctDNA to detect molecular residual disease (MRD) in patients with curatively resected CRC. Intriguingly, it has been reported that positive MRD based on ctDNA at the point of 4 weeks after surgery was a strong predictive marker in patients with stage II or III CRC. The recurrence rate with positive MRD was significantly higher compared to negative MRD (61.4% vs. 9.5%; HR, 10.0; *p* < 0.0001), and 18-month DFS was lower (38.4% vs. 90.5%) across all pathological stages. Furthermore, MRD-positive patients with high-risk stage II or stage III demonstrated a significant benefit from adjuvant chemotherapy (adjusted HR, 6.59; *p* < 0.001). These results indicate that MRD status based on ctDNA could stratify patients at postsurgical risk of recurrence and it can be useful information for deciding to conduct adjuvant chemotherapy [6].

## 4. Future Perspective

### Targeting Undruggable KRAS

*RAS* is a small GTPase that functions as a molecular switch to regulate proliferation, differentiation, and survival. Mutations in three *RAS* genes (*HRAS*, *NRAS*, and *KRAS*) and various other components of the *KRAS* signaling pathways are among the most common genetic alterations in human cancers, including 25% of lung, 40% of colorectal and 95% of pancreatic cancer [64]. However, despite its significance in cancer development, RAS protein had not resulted in any type of therapeutic attack, largely due to our incomplete understanding of how *RAS* is regulated and how it has been dismissed as “undruggable”. Finally, the development of small molecules that irreversibly bind to a common oncogenic mutant, *KRAS^G12C^*, was reported [8]. The inhibitor of *KRAS^G12C^*, sotorasib (AMG510) was first reported to show an anti-tumor effect and lead to the regression of *KRAS^G12C^*-mutated tumors [65]. The overall response rate (ORR) observed in patients with *KRAS^G12C^*-mutated non-small cell lung cancer (NSCLC) treated with sotorasib was 36% and sotorasib was first approved for *KRAS^G12C^*-NSCLC by FDA in 2021 [66]. More recently, adagrasib (MRTX849) has been approved with clinical efficacy (ORR 42.9%) for patients with *KRAS^G12C^*-NSCLC [67,68]. The frequency of *KRAS^G12C^* mutation in CRC is reported to be3~4% [64], however, most patients are refractory to sotorasib or adagrasib monotherapy, suggesting that a combination approach is required for better response [69]. Interestingly, it has been reported *KRAS^G12C^* demonstrated higher rates of basal EGFR activation compared with other *KRAS*-mutated CRC [70]. Thus, the dual inhibition of KRAS^G12C^ and EGFR could be a reasonable strategy. Adagrasib in combination with cetuximab showed that ORR was 46% (13/28) and DCR was 100% (28/28) in KRYSTAL-I study [71]. Also recently, a combination with sotorasib and panitumumab showed that ORR was 30% (12/40) with a DCR of 93% from CodeBreaK101 [72]. Another novel strategy is a combination with the inhibition of protein tyrosine phosphatase SHP2 that promotes the MAPK signaling pathway and is an essential factor in RAS-driven oncogenesis [64,73,74]. This combination therapy might enhance the anti-tumor activity and conquer the adaptive resistance to sotorasib monotherapy (Figure 1D) [65,75]. In a recent study, the anti-tumor efficacy of MRTX1133, a potent, selective, and non-covalent KRAS^G12D^ inhibitor was reported [76]. As the frequency of G12D mutation in *KRAS* is higher compared to G12C mutation in CRC, the potential impact of this drug is striking. More recently, feedback activation of EGFR restricts KRAS^G12D^ inhibitor efficacy in CRC, therefore, a combination therapy with KRAS^G12D^ and EGFR inhibitors for patients with *KRAS^G12D^*-mutated CRC could be promising [77].

## 5. Conclusions

The number of treatment options for mCRC patients has been growing. Especially, combination therapy could be useful to improve the prognosis of this heterogeneous disease. In this review, we have overviewed the combined systemic therapy as a standard of care for mCRC with targeting therapy including immunotherapy and introduced the future perspective for new drugs that target formerly undruggable genes. It is also worth noting that targeting a single gene or mutation might not be sufficient for eliminating the disease as cancer cells might harbor numerous genetic mutations. The combination strategy will be a promising approach to overcome the insufficient efficacy or resistance by monotherapy.
